# Non-coding RNAs in heart failure: epigenetic regulatory mechanisms and therapeutic potential

**DOI:** 10.3389/fgene.2025.1677797

**Published:** 2025-11-06

**Authors:** Yubo Ren, Bomeng Zhao, Luo Lv, Jingyuan Yang, Xiangting Nan, Bao Li, Bin Yang

**Affiliations:** 1 Second College of Clinical Medicine, Shanxi Medical University, Taiyuan, Shanxi, China; 2 The First College of Clinical Medicine, Shanxi Medical University, Taiyuan, Shanxi, China; 3 Shanxi Medical University, Taiyuan, Shanxi, China; 4 Department of Cardiology, The Second Hospital of Shanxi Medical University, Taiyuan, China; 5 School of Medicine, Shanxi Medical University, Taiyuan, China

**Keywords:** heart failure, non-coding RNA, epigenomics, microRNAs, long non-coding RNA, circular RNA, therapeutics

## Abstract

Heart failure represents the terminal stage of diverse cardiovascular disorders and continues to impose a heavy global burden despite advances in therapy. Beyond classical neurohormonal and hemodynamic pathways, recent studies underscore the central role of non-coding RNAs in orchestrating epigenetic remodeling that drives hypertrophy, fibrosis, and cardiomyopathy. In this review, we synthesize evidence into an integrative “ncRNAs–epigenetics–cardiomyopathy” working model that connects upstream non-coding RNAs regulation with chromatin dynamics and downstream pathological remodeling. We integrate evidence showing how microRNAs, long non-coding RNAs, and circular RNAs reshape transcriptional networks through interactions with DNA methylation, histone, and RNA modifications. Differential non-coding RNAs signatures across heart failure phenotypes, comorbidities, and complications are further highlighted, underscoring their potential utility in patient stratification and biomarker discovery. We also evaluate therapeutic frontiers that extend beyond single-target interventions toward multi-layered approaches, including antisense oligonucleotides, CRISPR/dCas9-mediated epigenome editing, and exosome- or nanoparticle-based delivery systems. Although translational barriers remain considerable, especially in terms of specificity, safety, and clinical validation, these strategies illustrate the potential of targeting the ncRNAs–epigenetic axis to advance precision medicine in heart failure.

## 1 Introduction

Heart failure (HF) is a chronic, debilitating syndrome and the final outcome of many cardiovascular disorders (Bozkurt et al., 2023; Savarese et al., 2023). Even with modern therapies, the 5-year survival of HF is about 50%, emphasizing the need for novel insights and strategies (Bozkurt et al., 2023; Shahim et al., 2023). This clinical challenge is compounded by the heterogeneity of HF. Multi-omics studies show that HF consists of distinct subtypes with associated comorbidities. These groups display different molecular signatures and regulatory pathways, which contribute to their variable responses to therapy (Lteif et al., 2024).

This diversity both supports molecular stratification and challenges the identification of unifying mechanisms (Metra et al., 2023). Non-coding RNAs (ncRNAs) are key regulators of cardiac signaling and remodeling, impacting gene expression at both transcriptional and post-transcriptional levels (Jalink et al., 2023; Mably and Wang, 2024). Parallel to this, epigenetic mechanisms, including DNA methylation, histone modifications, and chromatin remodeling, play central roles in shaping transcriptional programs during the progression of HF (Desiderio et al., 2024; Ho et al., 2024). Although each field has produced important insights, the interplay between ncRNAs and epigenetic mechanisms in driving cardiomyopathic remodeling remains poorly understood (Hajdarpašić et al., 2025). Although several authoritative reviews have advanced understanding of ncRNAs and epigenetic mechanisms in HF, a comprehensive model that clearly maps their interactions is still lacking (Lteif et al., 2024). Moreover, how these pathways relate to clinical subtypes and comorbidity profiles remains unclear, which limits their translational value (Foinquinos et al., 2020).

To address this gap, we organize current evidence into an integrative “ncRNAs–epigenetics–cardiomyopathy” regulation framework that explicitly links upstream ncRNAs regulation, intermediate epigenetic control, and downstream remodeling. We summarize how specific ncRNAs interact with epigenetic partners to modify chromatin states and transcriptional programs that drive pathological remodeling (Ho et al., 2024). We further discuss how these mechanisms may diverge across HF subtypes and comorbidity contexts, integrating evidence from molecular stratification studies (Versnjak et al., 2025).

We aim to integrate evidence across the “ncRNAs–epigenetics–cardiomyopathy” axis into a coherent, testable model and align it with HF phenotypes, comorbidities, and therapeutic targets. In addition to mechanistic insights, we emphasize emerging therapeutic strategies, including microRNAs (miRNAs) antagomirs (Täubel et al., 2021), long non-coding RNAs (lncRNAs)- and circular RNAs (circRNAs)-based interventions *via* exosome delivery (Reiss et al., 2023), and CRISPR/dCas9-driven epigenome editing (Liu and Olson, 2022). Together, these strategies may provide feasible approaches for developing individualized therapies in HF.

## 2 Epigenetic landscape of heart failure

### 2.1 DNA methylation

DNA methylation, the addition of a methyl group to cytosine at the 5-position, is a key epigenetic modification that regulates gene expression and contributes to pathological processes in HF, including hypertrophy and fibrosis (Liu and Tang, 2019). In HF animal models, genes such as GATA4 and MMP9 display marked changes in DNA methylation (Ho et al., 2024). These alterations contribute to the progression of ventricular remodeling. DNA methylation is crucial in the initiation and progression of myocardial hypertrophy and fibrosis. Abnormal DNA methylation of genes related to fibrosis, hypertrophy, and inflammation can drive maladaptive remodeling of the myocardium, a key precursor to HF. Abnormal expression of DNA methyltransferases (DNMTs), such as DNMT1 and DNMT3a, has been associated with HF, with changes in enzyme activity driving disease progression (Wu et al., 2020). NcRNAs can affect DNA methylation by regulating DNMT expression, thus altering gene activity and promoting myocardial fibrosis and hypertrophy. Consequently, inhibiting DNMTs could offer a potential therapeutic strategy to reverse myocardial injury (Garitano et al., 2025). However, substantial clinical evidence is still needed to establish their efficacy and safety (Garitano et al., 2025).

### 2.2 Histone modifications

Histone modifications regulate myocardial hypertrophy and apoptosis in HF. Histone deacetylases (HDACs) and histone acetyltransferases (HATs) control gene transcription by altering chromatin accessibility (Ooi et al., 2015). Histone acetylation and methylation regulate genes involved in myocardial hypertrophy, fibrosis, and inflammation. By activating or silencing pro-fibrotic targets, these modifications shape the course of heart failure (Xu et al., 2022). In animal models, HDAC inhibitors derived from these mechanisms lessen myocardial hypertrophy and prevent functional decline (Ooi et al., 2015; Han et al., 2022). Histone methylation, especially H3K27me3 and H3K4me3, influences cardiomyocyte proliferation and apoptosis. These changes reprogram myocardial transcription and drive heart failure development (Qi et al., 2025). NcRNAs influence histone modifications through their effects on histone-modifying enzymes. These changes contribute to myocardial fibrosis and hypertrophy.

### 2.3 RNA modifications

RNA modifications, especially N6-methyladenosine (m6A), are emerging as important regulators of myocardial hypertrophy (Li et al., 2022). M6A, a prevalent and tightly controlled RNA modification, occurs at the N6 position of adenosine residues. Mechanistically, m6A deposition is catalyzed by methyltransferase complexes such as METTL3, while its removal is mediated by demethylases such as FTO (Dorn et al., 2019; Berulava et al., 2020). m6A modulates transcriptional responses in stressed cardiomyocytes by controlling mRNA stability, splicing efficiency, and translation initiation (Choy et al., 2022). M6A regulates transcriptional responses in stressed cardiomyocytes by modulating mRNA stability, splicing efficiency, and translation initiation (Hinger et al., 2021). In HF, expression of m6A regulators is frequently dysregulated. These changes contribute to cardiomyocyte apoptosis and the activation of inflammatory responses (Choy et al., 2022; Shen et al., 2022).

### 2.4 Chromatin remodeling and nucleosome conformational changes

Chromatin remodeling modulates gene expression by altering nucleosome positioning and structure. In HF, altered activity of chromatin remodeling complexes, like the SWI/SNF complex, results in the reprogramming of cardiomyocyte transcriptional networks (Bevilacqua et al., 2014; Singh et al., 2023). NcRNAs interact with chromatin remodeling complexes, thereby modulating gene expression (Shi et al., 2022; Hao et al., 2025). These interactions contribute to myocardial remodeling in HF (Bevilacqua et al., 2014; Shi et al., 2022). Disruption of SWI/SNF function impairs the activation of repair-associated genes. This defect aggravates cardiac remodeling and accelerates the loss of function (Maassen et al., 2025).

### 2.5 Synergistic regulation by epigenetic modifiers and transcription factors

The progression of HF is coordinated by both epigenetic regulators and transcription factors. Among them, NF-κB and AP-1 initiate inflammatory and apoptotic signaling through DNA binding. These transcription factors are themselves regulated by epigenetic enzymes, including HDACs and HATs (Shi et al., 2022). NcRNAs modulate transcription factors and epigenetic regulators, through this regulation, they enhance inflammatory and apoptotic signaling, which promotes the progression of HF. For instance, the synergistic interaction between NF-κB and HDAC3 enhances myocardial inflammation, thereby accelerating HF progression (Zhang et al., 2024).

## 3 Non-coding RNA-mediated epigenetic regulation mechanisms in heart failure

### 3.1 Interaction between miRNAs and epigenetic enzymes

**FIGURE 1:**
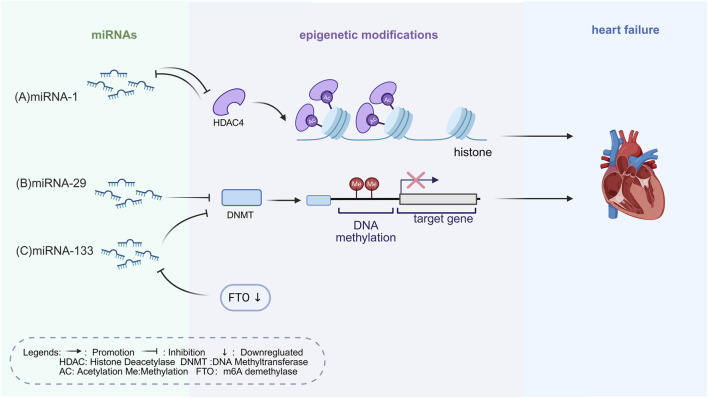
Epigenetic Mechanisms of miRNAs Regulation in HF. **(A)** MiR-1 targets HDAC4, blocking histone deacetylation to inhibit cardiac hypertrophy and alleviate HF. HDAC4 also negatively regulates the transcription of miR-1. **(B)** MiR-29b targets DNMT3a, blocking abnormal collagen gene methylation and inhibiting myocardial fibrosis. **(C)** MiR-133 targets DNMT3b to inhibit myocardial hypertrophy. FTO downregulation alters the function of miR-133a through m6A modification, weakening its ability to restrain myocardial hypertrophy and preserve cardiac function. The figure is created in https://BioRender.com.

MiRNAs are short non-coding RNAs of about 20–25 nucleotides. They regulate genes involved in HF by binding to the 3′ untranslated regions of target mRNAs, leading to their degradation or translational repression (Fumarulo et al., 2025). MiRNAs are central to the “ncRNAs–epigenetics–cardiomyopathy” framework. MiRNAs influence gene expression both directly and through interactions with other ncRNAs, contributing to the epigenetic remodeling observed in HF (Figure 1). Their regulatory effects involve targeting enzymes such as DNMTs, HDACs, and HATs, which subsequently modulate hypertrophy, fibrosis, apoptosis, and inflammatory responses in the myocardium (Shi et al., 2022).

For instance, miR-1 inhibits myocardial hypertrophy by targeting HDAC4, thereby limiting histone deacetylation (García-Padilla et al., 2024). Together, miR-1 and HDAC4 form a negative feedback loop, in which HDAC4 in turn suppresses the transcription of miR-1 (García-Padilla et al., 2024). This feedback loop represents an epigenetic regulatory mechanism in cardiomyopathy. During myocardial injury, disruption of this loop, characterized by reduced miR-1 and increased HDAC4, exacerbates disease progression, highlighting both the therapeutic potential and the risks of manipulating these pathways. Restoring miR-1 expression experimentally has been shown to attenuate myocardial hypertrophy and apoptosis (Greco and Condorelli, 2015; García-Padilla et al., 2024). Nevertheless, findings on miR-1 levels after ischemia–reperfusion remain contradictory: some studies describe transient downregulation that normalizes within a week, whereas others report upregulation. These discrepancies likely reflect heterogeneity in experimental models, reperfusion protocols, tissue sources, and cell populations (Pan et al., 2012). Consequently, the therapeutic promise of miR-1 appears context-dependent and requires cautious evaluation.

MiR-21 exerts opposing effects depending on cell type. In cardiomyocytes, it limits apoptosis by inhibiting pro-apoptotic pathways such as PTEN/PDCD4, thereby mitigating myocardial injury (Kura et al., 2020). In contrast, in cardiac fibroblasts, miR-21 suppresses TGF-β_1_ receptor III and activates the TGF-β_1_ pathway, which enhances collagen deposition and contributes to HF progression *via* fibrosis (Khalaji et al., 2024). Moreover, exosome-delivered miR-21 has been shown to regulate TGF-β_1_ translation, further influencing fibrotic signaling (Xue et al., 2020). These findings highlight the dual and sometimes conflicting roles of miR-21—protective in reducing cardiomyocyte apoptosis but deleterious in driving fibroblast-mediated fibrosis. Clarifying the disease- and cell-specific contexts of miR-21 activity will be essential for developing safe and effective miR-21–targeted therapies (Huang et al., 2024; Alabere et al., 2025). Quantitative, context-resolved studies are needed to disentangle these cell-type specific roles, ideally by combining transcriptomic, proteomic, and spatial data.

MiR-133 suppresses myocardial hypertrophy by downregulating DNMT3b expression and inhibiting DNA methylation (Chavali et al., 2012). MiR-133 expression is upregulated in exosomes secreted by hypoxia/reoxygenation-treated endothelial progenitor cells. Mediated by YBX-1, miR-133 is packaged into exosomes and transferred to cardiac fibroblasts, promoting angiogenesis and mesenchymal-endothelial transition, alleviating myocardial fibrosis (Lin et al., 2019). MiR-133 regulates cardiac remodeling by affecting epigenetic mechanisms, including DNA methylation and histone modification. It acts on hypertrophy and fibrosis, which are major contributors to HF. In a mouse model of diabetic cardiomyopathy, overexpression of miR-133a improved structural pathology but failed to restore diastolic function, as determined by pressure-volume loop measurements (Kambis et al., 2019). These results suggest that the protective effects of miR-133a arise mainly from its inhibition of fibrosis and hypertrophy, rather than from direct modulation of diastolic function.

A study using a mouse model and human serum samples integrated transcriptome, methylation, and miRNA sequencing data to explore miRNA roles in HF. It identified FTO and IGF2BP2 as key m6A methylases, with altered expression in pressure-overload induced HF mice (Wang et al., 2025). In HF, reduced expression of FTO enhances m6A modification on RNA. This epigenetic alteration, mediated by reader proteins such as IGF2BP2, disrupts the cardioprotective role of miR-133a, thereby weakening its ability to restrain myocardial hypertrophy and preserve cardiac function (Mathiyalagan et al., 2019; Qian et al., 2021). Although not a classical chromatin-modifying enzyme, m6A regulators such as FTO also fall under the broader scope of epigenetic control, thereby linking RNA epigenetics with miRNA function.

In cardiac fibroblasts, miR-29b targets DNMT3a, blocking aberrant collagen gene methylation and thereby inhibiting myocardial fibrosis (Qin et al., 2018). Notably, miR-29 alterations have been reported in human end-stage failing hearts and in circulation from patients with hypertrophy/fibrosis, while animal models sometimes show divergent expression patterns—underscoring the need to validate ncRNA-based therapies in human HF tissues and contexts (van Rooij et al., 2008; Liu et al., 2019).

Although miRNAs hold promise as biomarkers, their expression variability and complex regulation at different stages of heart failure limit their current clinical applicability as reliable biomarkers. More research is needed to develop standardized protocols for miRNA measurement and ensure accuracy across diverse patient groups.

Recent studies indicate that a subset of nuclear microRNAs/Argonaute proteins complexes can interact with chromatin or promoter regions, thereby recruiting or associating with chromatin-modifying enzymes such as HDACs and DNMT3 to regulate transcriptional activity (Billi et al., 2024). In certain genomic contexts, these interactions may facilitate the enrichment of repressive epigenetic marks such as H3K27me3 or DNA methylation, promoting a local transition from an active chromatin configuration to a silent heterochromatin state (Billi et al., 2024; Jiang et al., 2024). However, targeting such processes for therapeutic purposes remains challenging, since perturbation of nuclear miRNA functions may disrupt essential regulatory networks and provoke off-target effects (Jiang et al., 2024). These considerations highlight the complexity of selecting suitable molecular targets for miRNA-based therapies while minimizing risks of dysregulation (Kim and Croce, 2023).

### 3.2 lncRNAs and chromatin-modifying complexes

LncRNAs, typically over 200 nucleotides, regulate heart failure by interacting with DNA, RNA, and proteins to influence hypertrophy and fibrosis (Zheng et al., 2021; Fan et al., 2022). Within the “ncRNAs–epigenetics–cardiomyopathy” framework, lncRNAs serve as regulators. Acting as competing endogenous RNAs (ceRNAs), they bind miRNAs and release target genes from repression, thereby contributing to epigenetic regulation in heart failure (Kalozoumi et al., 2014). Lacking protein-coding capacity, they act as scaffolds to position chromatin-modifying complexes and transcription factors at specific genomic regions (Razin and Gavrilov, 2021). This process alters chromatin architecture to either activate or repress gene expression (Shi et al., 2022; Mably and Wang, 2024).

LncRNA Cardiac Hypertrophy Associated Epigenetic Regulator (chaer) serves as an early epigenetic checkpoint (Wang et al., 2021). In cardiomyocytes, chaer directly binds to the core catalytic subunit EZH2 of PRC2, disrupting its recruitment to pro-hypertrophic gene promoters such as β-MHC/MYH7 (Lizcano and Bustamante, 2022). This reduces H3K27me3 deposition, activates hypertrophic genes, and promotes myocardial hypertrophy (Wang et al., 2021).

Some lncRNAs also function as miRNA precursors. For example, upon binding to the PRC2 complex, H19 inhibits H3K27me3 deposition at the promoter of the antihypertrophic gene Tescalcin, which downregulates the NFAT signaling pathway, thereby preventing and reversing pathological myocardial hypertrophy (Viereck et al., 2020). In addition, H19 also encodes miR-675, which indirectly inhibits myocardial hypertrophy (Li et al., 2023; Li et al., 2024; Mably and Wang, 2024).

LncRNA Metastasis-Associated Lung Adenocarcinoma Transcript 1 (MALAT1) influences SR-protein phosphorylation and thereby modulates alternative splicing and angiogenic programs. Similar mechanisms contribute to cardiac fibrosis and HF (Huang et al., 2019). As a ceRNA, MALAT1 sequesters miR-145, and H19 sequesters miR-29b, both of which relieve the inhibition of the TGF-β_1_ pathway, thereby promoting collagen deposition and myocardial fibrosis (Huang et al., 2019; Guo et al., 2022). The role of H19 appears bidirectional depending on molecular interactions. LncRNAs might serve as precision guides that target SWI/SNF to specific genomic sites to control chromatin structure and gene expression (Oo et al., 2025).

Beyond chromatin-level epigenetics, lncRNAs are also regulated by RNA modifications such as m6A, which indirectly influence chromatin state and cardiac remodeling. Recent studies highlight that m6A modification in lncRNAs is key for regulating HF through epigenetic pathways. m6A, the most common RNA mark, is added to certain adenosines by enzymes such as METTL3, METTL14, and WTAP. One example is MIAT, which contributes to Angiotensin II-induced cardiac hypertrophy. The m6A reader YTHDF2 binds MIAT and controls fatty acid metabolism genes like CPT-1a and PPARα, thereby affecting cardiac growth (Yang et al., 2022). These findings point to the lncRNA–m6A–metabolism axis as a driver of cardiac remodeling.

In myocardial fibrosis, METTL3 increases the m6A mark YTHDF2 then drives its degradation. This promotes mitochondrial fission, fibroblast activity, and collagen build-up, which worsen fibrosis and HF (Tu et al., 2023). In the myocardial ischemia-reperfusion injury (MIRI) model, METTL3 increases m6A levels on lncRNA Small Nucleolar RNA Host Gene 8 (SNHG8), enabling it to bind PTBP1 and regulate ALAS2, thereby exacerbating oxidative stress and injury (Tang et al., 2023), thus promoting the occurrence and progression of HF following myocardial infarction. In ferroptosis and oxidative stress, lncRNA Urothelial Carcinoma Associated 1 (UCA1) boosts the demethylase FTO, lowers m6A, and enhances NRF2 expression, helping protect heart cells (Jiao et al., 2024).

Conversely, in HF models, bone marrow mesenchymal stem cell–derived exosomes can replenish lncRNA Growth Arrest Specific (GAS5) levels in cardiomyocytes. Restored GAS5 epigenetically upregulates UL3, activates Hippo signaling effectors YAP and TAZ, and blocks ferroptosis, thereby mitigating fibrosis and slowing pathological remodeling (Ren and Zhao, 2024). These findings indicate that GAS5 exerts opposite effects depending on its regulatory context—being inactivated by m6A-dependent degradation yet restored by exosomal delivery—highlighting the hallmark context dependence of lncRNAs biology and the need for rigorous multi-omics validation.

LncRNAs play an important role in chromatin remodeling and nucleosome structure, processes that are central to the development of heart failure (Fan et al., 2022; Xie et al., 2025). Through interactions with chromatin remodeling factors, lncRNAs also shift nucleosome positioning, changing gene accessibility and transcription. Such chromatin-related actions of lncRNAs are strongly linked to maladaptive myocardial remodeling in HF.

In addition to chromatin and RNA modification pathways, lncRNAs delivered by exosomes also modulate HF through ceRNA interactions. Exosomes from human mesenchymal stem cells also carry lncRNA Kruppel-like factor 3 antisense RNA 1(KLF3-AS1), which promotes cardiomyocyte survival. By sponging miR-138-5p and raising Sirt1 levels, KLF3-AS1 reduces apoptosis and protects against ischemia/reperfusion injury, limiting the progression of heart failure (Mao et al., 2019). Since MIRI injury are major drivers of HF progression, these findings suggest a potential role for exosomal lncRNAs in HF therapy, although further validation in chronic HF models is still required (Jha et al., 2023).

**FIGURE 2:**
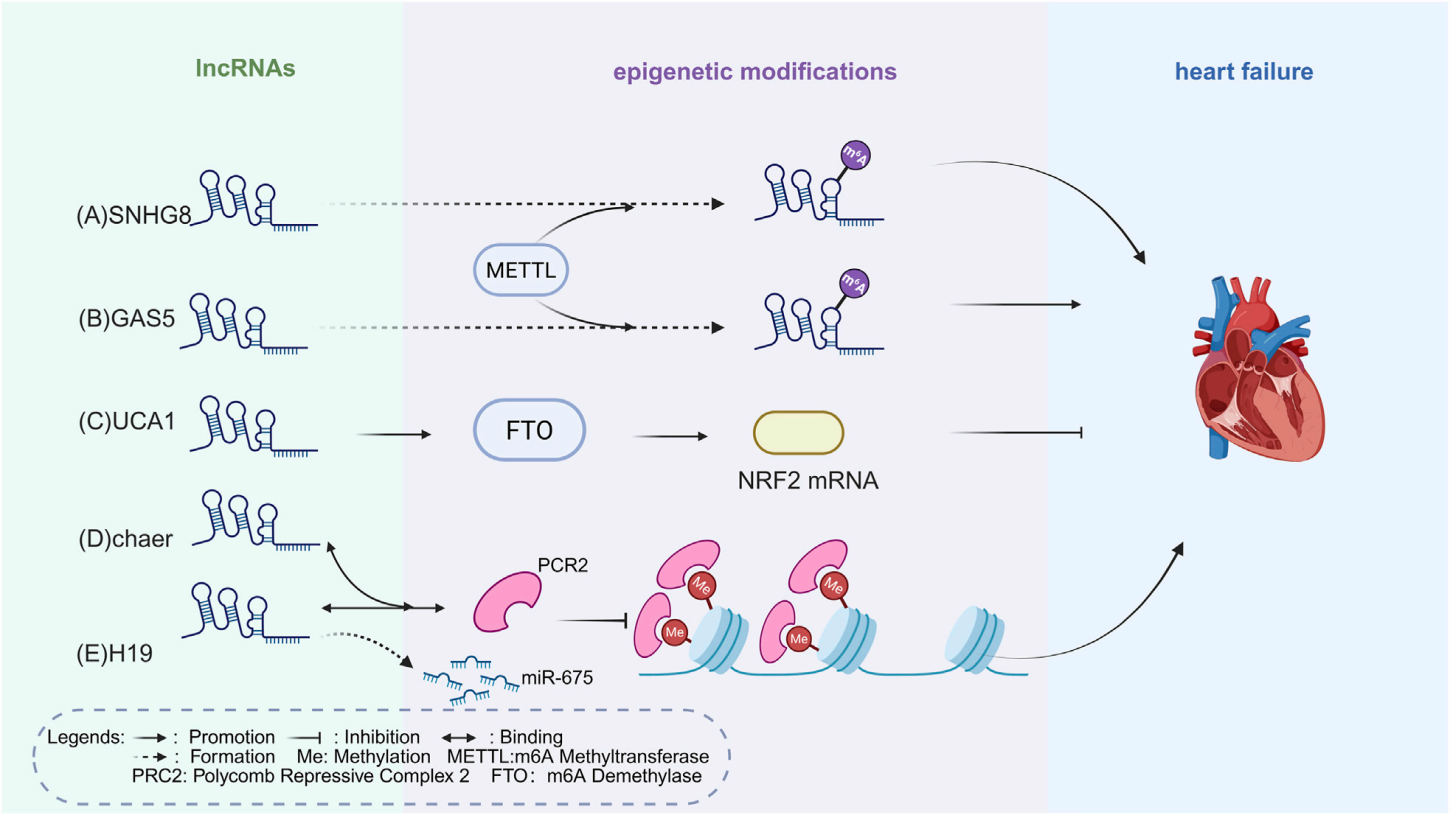
Epigenetic Mechanisms of lncRNAs Regulation in HF. **(A)** METTL3 enhances m6A levels on lncRNA SNHG8, which in turn exacerbates oxidative stress and injury, thereby driving the onset and progression of heart failure post-myocardial infarction. **(B)** METTL3 exacerbates myocardial fibrosis by enhancing the m6A modification of lncRNA GAS5, ultimately contributing to HF. **(C)** LncRNA UCA1 upregulates the expression of the demethylase FTO, thereby enhancing NRF2 mRNA expression, protecting cardiomyocytes from oxidative stress, and delaying the progression of HF. **(D)** LncRNA chaer directly binds with PRC2 to inhibit histone methylation on hypertrophic gene promoters, promoting cardiac hypertrophy and contributing to HF. **(E)** LncRNA H19 binds to PRC2, resulting in the demethylation of hypertrophic and collagen genes, thus promoting myocardial hypertrophy and fibrosis, and contributing to HF. H19 also encodes miR-675, which indirectly inhibits myocardial hypertrophy. The figure is created in https://BioRender.com.

These processes highlight the central role of lncRNAs in the “ncRNAs–epigenetics–cardiomyopathy” model, where lncRNAs control gene expression *via* classical epigenetic mechanisms, contributing to heart failure pathogenesis (Figure 2). However, targeting these interactions therapeutically is complex, as the role of specific lncRNAs in chromatin dynamics depends on context. Despite promising findings, further investigation is needed to better understand these processes and develop more precise and effective therapies.

### 3.3 Roles of circRNAs in epigenetic regulation

**FIGURE 3:**
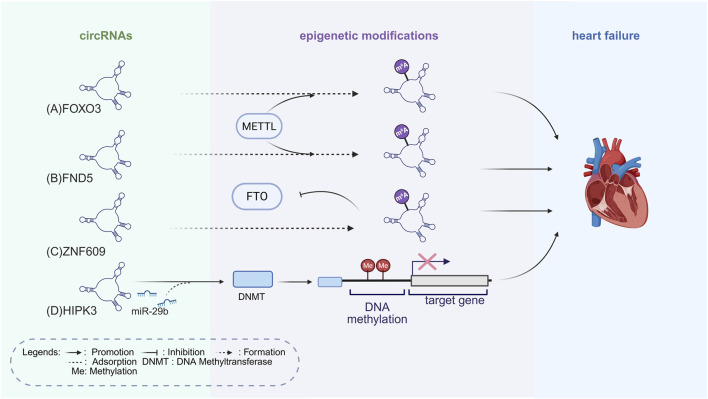
Epigenetic Mechanisms of circRNAs Regulation in HF. **(A)** CircRNA FOXO3 undergoes m6A modification mediated by METTL3, which enhances its stability and promotes myocardial apoptosis, thereby accelerating HF progression. **(B)** M6A-modified circRNA FND5, mediated by METTL3, regulates oxidative stress in cardiomyocytes and accelerates HF progression. **(C)** CircRNA ZNF609 is stabilized by m6A modification, and its accumulation suppresses FTO, maintaining elevated m6A levels that exacerbate cardiomyocyte injury and HF progression. **(D)** CircRNA HIPK3 adsorbs miR-29b-3p, relieving its suppression of DNMT3a, increasing DNA methylation levels, activating cardiac fibroblasts, exacerbating cardiac fibrosis, and worsening heart failure. The figure is created in https://BioRender.com.

CircRNAs, characterized by a covalently closed loop structure, are highly stable and act primarily as “miRNA sponges” in HF, sequestering miRNAs to relieve repression of target genes and epigenetic regulators, thereby influencing cardiomyocyte function (Lan et al., 2024). By functioning both as regulators and targets within the “ncRNAs–epigenetics–cardiomyopathy” network, circRNAs serve as key mechanistic links connecting epigenetic modulation to cardiomyopathy progression (Figure 3). They interact with RNA-binding proteins to regulate RNA splicing and stability, making them potential therapeutic targets in HF (Jiang et al., 2020; You et al., 2025). For example, circRNA HIPK3 sponges miR-29b, promoting collagen expression and fibroblast activation (Zhang et al., 2021). CircRNA HIPK3 functions as a sponge for miR-185-3p, leading to the upregulation of the calcium-sensing receptor (CaSR) (Zhang et al., 2021). Elevated CaSR expression increases cardiomyocyte Ca^2+^ sensitivity and amplifies downstream phospholipase C (PLC), inositol 1,4,5-trisphosphate (IP_3_) signaling, which in turn drives cardiomyocyte hypertrophy (Zhang et al., 2021). Because of the pro-fibrotic effects, circRNA HIPK3 is considered a critical clinical intervention target (Zhang et al., 2021).

Recent studies point to an important role of m6A-modified circRNAs in HF. CircRNA Forkhead box O3 (FOXO3), for example, becomes more stable after m6A modification (mediated by METTL3) and promotes myocardial apoptosis through the p53/p21 pathway, which accelerates HF progression (Sun et al., 2021). Its involvement in other cardiac conditions also remains largely unexplored and requires more study.

CircRNA Zinc Finger Protein 609 (ZNF609) becomes more stable through m6A modification and accumulates under Doxorubicin (DOX)-induced cardiotoxicity, where it suppresses the demethylase FTO and sustains elevated m6A levels (Yu et al., 2023). Its upregulation promotes cardiomyocyte apoptosis, Reactive Oxygen Species (ROS) production, and fibrosis, while knockdown alleviates injury and improves cardiac function. The findings are limited to an acute DOX model; validation in chronic heart failure and the downstream m6A targets remain unresolved (Yu et al., 2023).

M6A-modified circRNA Fnd5, mediated by METTL3, regulates oxidative stress in cardiomyocytes and accelerates heart failure progression (Jiang et al., 2025). Existing data are encouraging but point to the need for broader studies to clarify the functions of m6A-modified circRNAs in heart failure. Current findings remain scattered and inconsistent, and more work is needed to define their impact on cardiac disease.

Certain circRNAs bind directly to histone-modifying complexes or RNA polymerase complexes, altering gene transcription (Yuan H. et al., 2024). RNA sequencing of plasma exosomal circRNAs in patients with dilated cardiomyopathy and chronic heart failure identified 49 with altered expression. These circRNAs may contribute to disease progression and serve as biomarkers. The study was limited by small sample size and patient number, restricting generalizability. The cardiac origin of circulating circRNAs and the role of exosomal nucleocytoplasmic regulators in chronic heart failure require further study (Xu et al., 2024).

Taken together, circRNAs, like lncRNAs, exhibit context-dependent roles in epigenetic regulation. By acting as miRNA sponges, interacting with chromatin-modifying complexes, and undergoing m6A-dependent modulation, circRNAs add an additional regulatory layer to cardiac remodeling. When integrated with miRNAs and lncRNAs, they form a multi-layered “ncRNAs–epigenetics–cardiomyopathy” framework that highlights both commonalities and distinct regulatory features across ncRNA classes. Different ncRNAs show distinct features in their regulatory mechanisms and functional roles (Table 1). In summary, this “ncRNAs–epigenetics–cardiomyopathy” regulatory framework illustrates the mechanisms of action from the molecular, cellular, and tissue levels (Table 2). This framework forms a crucial link between ncRNAs and the fundamental pathological mechanisms of HF, providing valuable insights for advancing precision medicine in its treatment.

**TABLE 1:** Classification and functional overview of ncRNAs.

ncRNA	Length	Structural features	Functions	References
miRNA	Approximately 20–25 nts	Single-stranded	Targeting mRNA to inhibit translation	Kalozoumi et al. (2014)
lncRNA	Greater than 200 nts	Linear structure	Regulation of transcription and chromatin state	Mably and Wang (2024)
circRNA	Averagely 500 nts	Circular structure	Adsorption of miRNA	Hansen et al. (2013), Memczak et al. (2013)

Abbreviation: nts, nucleotides.

**TABLE 2:** Regulatory roles of ncRNAs in myocardial hypertrophy and myocardial fibrosis.

ncRNA	Targeted enzyme/molecule	Mechanistic action	Cardiac hypertrophy	Cardiac fibrosis	Myocardial oxidative stress	Myocardial apoptosis	References
miR-1	HDAC4	Blocking histone deacetylation	↓				Greco and Condorelli (2015), García-Padilla et al. (2024)
miR-21	TGF-β_1_ receptor III	Relief of TGF-β_1_ pathway inhibition		↑			Liu et al. (2010), Kura et al. (2020)
miR-29b	DNMT3a	Inhibition of collagen gene methylation		↓			Qin et al. (2018)
miR-133	DNMT3b	Inhibition of DNA methylation	↓				Chavali et al. (2012)
	FTO	FTO downregulation alters the function of miR-133a through m6A modification	↑				Qian et al. (2021)
lncRNA chaer	PRC2	Blocking EZH2 recruitment to Genes	↑				Wang et al. (2016)
lncRNA H19	PRC2	Inhibition of H3K27me3 methylation	↑				Viereck et al. (2020)
	miR-29b	Relief of TGF-β_1_ pathway inhibition		↑			Guo et al. (2022)
lncRNA MALAT1	miR-145	Relief of TGF-β_1_ pathway inhibition		↑			Huang et al. (2019)
lncRNA SNHG8	METTL3	Binding of PTBP1 and regulation of ALAS2			↑		Tang et al. (2023)
LncRNA UCA1	FTO	Reduction of m6A and enhancement of NRF2 expression			↓		Jiao et al. (2024)
circRNA HIPK3	miR-29b	Promotion of DNA methylation		↑			Zhang et al. (2021)
	miR-185	Upregulation of CaSR	↑				
lncRNA GAS5	METTL3	YTHDF2-mediated degradation and reduction of GAS5 levels		↑			Tu et al. (2023)
circRNA FOXO3	METTL3	regulation of the p53/p21 pathway				↑	Sun et al. (2021)
circRNA ZNF609	FTO	Suppression of the demethylase FTO and maintenance of elevated m6A levels		↑	↑	↑	Yu et al. (2023)
circRNA FND5	METTL3	METTL3-mediated m6A modification			↑		Jiang et al. (2025)

Abbreviation: ↑, promotion, ↓, inhibition.

In the process of HF, miR-21 can both inhibit apoptosis pathways such as PTEN/PDCD4 to protect cardiomyocytes and activate fibroblasts, promoting myocardial fibrosis. However, its overall effect is primarily to promote myocardial fibrosis. The content of miR-675, encoded by lncRNA H19, is extremely low in adult myocardium, hence it is not listed.

## 4 Differential ncRNA expression profiles in different heart failure types, comorbidities and complications

This section explores the expression patterns and functional significance of ncRNAs in various types of heart failure, along with their common comorbidities and high-mortality complications (Figure 2; Table 3). The findings are expected to deepen our understanding of the pathogenesis of heart failure and offer valuable insights for clinical diagnosis and treatment strategies.

**TABLE 3:** Expression profiles of ncRNAs in different heart failure phenotypes and coexisting syndromes.

HF Types/HF with comorbidities/complications	ncRNA	Expression	Function/mening	References
HFrEF	miR-375	↓	Diagnosis of HFrEF	Watson et al. (2015)
	miR-208	↑	Inhibition of Myh7 expression	Montgomery et al. (2011)
	miR-423-5p	↑	Negatively correlated with LVEF	Guo et al. (2024)
	exo-miR-92b-5p	↑	Diagnosis of HFrEF	Wu et al. (2018)
	lncRNA SRA1	↑	Differentiation between HFpEF and HFrEF	Boichenko et al. (2025)
	lncRNA HEAT2	↑	Prediction of HFrEF occurrence and mortality	Boeckel et al. (2019)
	lncRNA GDE1-1:1	↓	Negatively correlated with LVEF	Qi et al. (2024)
	circRNA DEPCS	↑	Differentiation between HFpEF and HFrEF	Boichenko et al. (2025)
HFpEF	miR-19b-3p	↓	Differentiation between HFpEF and HFrEF	Parvan et al. (2022)
	miR-222/221	↑	Multidimensional regulation of myocardial cell homeostasis	Knyrim et al. (2021)
	lncRNA TUG1	↑	a potential biomarker	Boichenko et al. (2025)
	lncRNA MHRT	↑	Prediction of HF severity	Boichenko et al. (2025)
	lncRNA XIST	↑	Participation in cardiac remodeling	Xiao et al. (2019), Nie et al. (2023)
	circRNA HECW2	↑	Promotion of myocardial fibrosis	Boichenko et al. (2025)
HF with diabetes	lncRNA MALAT1	↑	Promotion of myocardial apoptosis and fibrosis	Wang et al. (2023)
	lncRNA HOTAIR	↓	Promotion of cardiac oxidative stress response	Qi and Zhong (2018)
	lncRNA KCNQ1OT1	↑	Promotion of cardiomyocyte pyroptosis and fibrosis	Yang et al. (2018)
	miR-34a	↑	Correlated with LVDF	Bernardo et al. (2022)
	miR-21	↓	Prediction of cardiac diastolic disorders	Dai et al. (2018)
HF with CKD	miR-21	↓	Correlated with renal function indicators and heart-failure severity	Wang et al. (2020)
HF with CAD	miR-132	↑	Promotion of myocardial remodeling and fibrosis	Täubel et al. (2021)
	miR-208a/β	↑	A supplementary biomarker to troponin	Huang et al. (2021)
	lncRNA ANRIL	↑	Exacerbation of plaque formation	Fasolo et al. (2019)
HF with Obesity	miR-122	↑	Promotion of cardiac hypertrophy and fibrosis	Wang et al. (2019)
	miR-33	↑	Participation in cardiovascular remodeling	Ortega et al. (2023)
HF with AF	lncRNA UCA1	↑	Promotion of Myocardial Hypertrophy	Boichenko et al. (2025)
	lncRNA SARRAH	↓(atrial tissue)	Resistance to oxidative stress and ischemic injury	Trembinski et al. (2020)
		↑(serum)		

Abbreviation: ↑, upregulated; ↓, downregulated.

### 4.1 Differential expression of ncRNAs in distinct heart failure phenotypes

**FIGURE 4:**
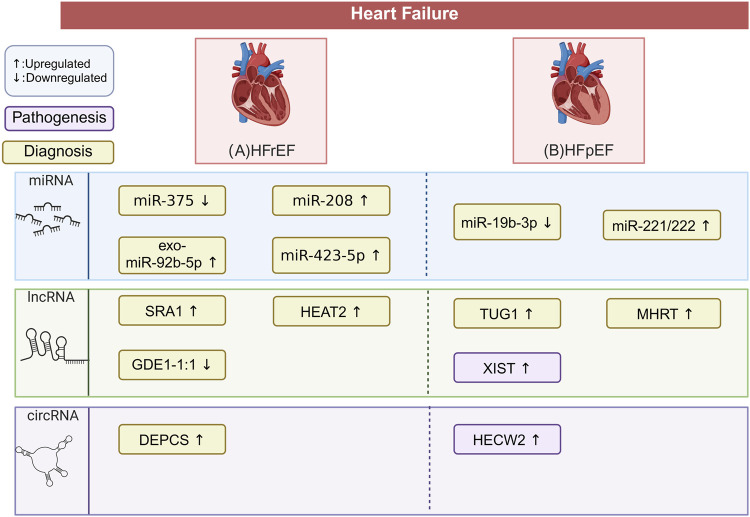
Differential ncRNA Expression in HF Types. This figure outlines the characteristic ncRNAs signatures across in patients with different types of HF. The pathogenesis and diagnoses depicted are all related to heart failure. The potential biomarkers mentioned require validation through large-scale, multicenter clinical trials. This does not imply that these biomarkers are without impact on the disease pathogenesis. **(A)** In HFrEF, miR-375 and lncRNA GDE1-1:1 are downregulated, while miR-208, miR-423-5p, exo-miR-92b-5p, lncRNA SRA1, lncRNA HEAT2, and circRNA DEPCS are upregulated. These molecules are potential biomarkers for HFrEF diagnosis. **(B)** In HFpEF, miR-19b-3p is downregulated, while miR-222/221, lncRNA TUG1, lncRNA MHRT, and circRNA HECW2 are upregulated. These molecules serve as potential biomarkers for HFpEF, with lncRNA XIST and circRNA HECW2 linked to profibrotic and inflammatory pathways. The figure is created in https://BioRender.com.

HF is a multifactorial syndrome typically classified into two main phenotypes based on left ventricular ejection fraction: heart failure with reduced ejection fraction (HFrEF, EF <40%), heart failure with preserved ejection fraction (HFpEF, EF ≥50%), and heart failure with mid-range ejection fraction (HFmrEF, 40% ≤EF ≤ 49%) (Hu et al., 2022). Within the framework of “ncRNAs–epigenetics–cardiomyopathy” network, these HF subtypes display distinct ncRNA expression profiles, revealing molecular heterogeneity at the epigenetic level. Through diverse epigenetic modifications, ncRNAs modulate gene expression and shape myocardial remodeling as well as disease progression (Figure 4). Such characteristics highlight ncRNAs as promising biomarkers, particularly valuable for subtype classification and prognostic assessment in the context of epigenetic regulation.

#### 4.1.1 ncRNA expression characteristics in HFrEF

In the peripheral blood of HFrEF patients, miR-375 is downregulated. The combined detection of this miRNA with B-type natriuretic peptide (BNP) significantly improves the diagnostic performance in distinguishing HFrEF from HFpEF (Watson et al., 2015; Lozano-Velasco et al., 2024). In adult hearts, miR-208 is critical for the expression of Myh7 (β-myosin heavy chain) (Saravanan et al., 2025). Plasma miR-423-5p is elevated in patients with HFrEF, showing a negative correlation with left ventricular ejection fraction (LVEF) and rising progressively with higher HF class (NYHA II–IV) (Guo et al., 2024). However, further clinical validation is necessary.

In HF patients, the expression level of lncRNA steroid receptor RNA activator 1 (SRA1) is significantly higher than that in healthy individuals (Boichenko et al., 2025). Moreover, SRA1 shows a positive correlation with BNP levels, left atrial diameter, and left ventricular end-diastolic diameter, while being negatively correlated with LVEF (Boichenko et al., 2025). SRA1 shows considerable potential in distinguishing HF patient subtypes. Compared to healthy controls, it can effectively identify patients with HFrEF (AUC = 0.891) (Yu et al., 2024). In the “ncRNAs–epigenetics–cardiomyopathy” axis, SRA1 is pro-fibrotic, promoting the activation of cardiac myofibroblasts by downregulating miR-148b (Zhang et al., 2019). Additionally, it can reduce hypoxia-induced damage in cardiomyocytes by modulating the PPARγ/NF-κB signaling pathway (Zhang et al., 2018). HEAT2 is upregulated in the blood of patients with HFrEF and holds potential as a biomarker,however, its predictive value for incidence and mortality requires validation in large prospective cohorts (Boeckel et al., 2019). In a cohort of maintenance hemodialysis patients, lncRNA GDE1-1:1 (ENST00000561762) was detectable when LVEF fell below 40%, with its expression showing a progressive decline as LVEF worsened, suggesting a negative association with HFrEF progression (Qi et al., 2024). However, given the small sample size and the specificity of the dialysis population, its generalizability and clinical utility require validation in large, multicenter cohorts including non-dialysis populations. Preliminary evidence has suggested that certain circRNAs, such as circR DEPCS, may be associated with serum BNP levels in HFrEF, although the supporting data remain limited and largely derived from small-scale studies summarized in review articles (Boichenko et al., 2025). The functional role of DEPCS in HFrEF pathogenesis therefore remains unclear and warrants further investigation (Boichenko et al., 2025).

Exosome-delivered miR-92b-5p (exo-miR-92b-5p) demonstrates high sensitivity and specificity for diagnosing HFrEF, suggesting that circulating levels of exo-miR-92b-5p can serve as a reliable diagnostic biomarker for the condition (Wu et al., 2018). However, as this evidence derives from a small, single-center study, validation in larger, multicenter cohorts is required.

Sex and aging influence this axis differently: estrogen-regulated miR-21 and X-linked miRNAs drive sex-specific remodeling (Queirós et al., 2013; Emerson et al., 2025), whereas aging downregulates protective promotes pro-fibrotic ones (miR-21), alongside epigenetic drift that creates a frail profile (Bei et al., 2018; Mak et al., 2023). These patterns highlight the importance of considering sex and age in biomarker design and therapeutic strategies.

Within the “ncRNAs–epigenetics–cardiomyopathy” framework, studies on HFrEF emphasize how ncRNAs modulate cardiomyocyte function and phenotypic transition through epigenetic mechanisms, thereby promoting remodeling and fibrosis (Deogharia and Gurha, 2024). Alterations in SRA1 (Boichenko et al., 2025), miR-375 (Booz and Theofilatos, 2022) and miR-133 (Ho et al., 2024) are implicated in of HFrEF progression. These molecules, together with exosomal miR-92b-5p, also hold promise as biomarkers for diagnosis and subtype classification (Boichenko et al., 2025).

Several hurdles remain before ncRNA biomarkers can be applied clinicall (Booz and Theofilatos, 2022). Results often lack reproducibility across different platforms and sample types, including plasma, serum, whole blood, and exosomes (Xie et al., 2025). Many ncRNAs, such as miR-21 and miR-423-5p, are not disease-specific, as they are also altered in other cardiovascular or systemic conditions (Ho et al., 2024).

#### 4.1.2 ncRNA expression characteristics in HFpEF

In HFpEF, miR-19b-3p expression is markedly downregulated, and the magnitude of this suppression effectively discriminates HFpEF from HFrEF (Parvan et al., 2022). Elevated peripheral blood lncRNA TUG1 (taurine upregulation 1) has been reported as a potential biomarker in HF, with possible relevance for HFpEF (Boichenko et al., 2025). Additionally, lncRNA H19 is highly expressed in HFpEF patients (Jalink et al., 2023). lncRNA myosin heavy chain-associated RNA transcripts (MHRTs) has been proposed as a potential predictor of HF severity (Boichenko et al., 2025). In one study, MHRT was upregulated in plasma samples from 72 HF patients compared to 60 non-HF control subjects (Xuan et al., 2017). Several circRNAs have been reported to be upregulated in HF and implicated in profibrotic and inflammatory pathways, with potential relevance to HFpEF (Boichenko et al., 2025).

Sex and age strongly influence the clinical features of HFpEF. Women show a higher prevalence and disease burden than men (May et al., 2025). These differences may be partly explained by molecular mechanisms influenced by sex hormones and X chromosome dosage, which have been shown to modulate DNA methylation and chromatin regulation (Knowlton and Lee, 2012). The “ncRNA–epigenetics–cardiomyopathy” axis is modulated by both age and sex. For example, the miR-222/221 cluster, regulated by estrogen signaling, influences electrophysiology, calcium homeostasis, and cellular proliferation in cardiac and vascular cells (Knyrim et al., 2021). Such mechanisms align with microvascular dysfunction and impaired exercise reserve commonly observed in women with HFpEF (Schulz et al., 2023).

The lncRNA X-inactive specific transcript (XIST), a female-specific epigenetic factor, has also been implicated in myocardial remodeling through chromatin modification and the regulation of inflammation- and fibrosis-related genes (Xiao et al., 2019; Nie et al., 2023). However, most of these findings come from animal models or small cohorts, and large-scale validation remains limited (May et al., 2025).

In the framework, age-related ncRNAs changes further contribute to HFpEF pathophysiology. miR-22 is upregulated in the aging myocardium, where it promotes fibroblast senescence (Jazbutyte et al., 2013). In patients with HFpEF, several lncRNAs, including ENST00000559220, are differentially expressed in epicardial adipose tissue and associated with inflammatory, oxidative stress, and immune pathways (He et al., 2024). Although direct links between these lncRNAs and diastolic function across age groups remain unclear, such findings highlight a potential role of age-related ncRNAs in HFpEF.

Collectively, ncRNA alterations driven by sex and aging may underlie key pathological features, including reduced myocardial compliance, inflammation, and microvascular dysfunction. Yet current evidence is fragmented, tissue- and model-dependent, and requires validation in larger, well-stratified clinical studies (Parvan et al., 2022). Patient variability related to age, sex, comorbidities, and genetics further complicates interpretation. Most studies so far rely on small, single-center cohorts, highlighting the need for large multicenter trials to define standardized cutoffs and assess added value beyond established biomarkers like BNP and N-terminal pro-B-type natriuretic peptide (NT-proBNP) (Meijers et al., 2017; Cediel et al., 2020).

#### 4.1.3 ncRNA expression characteristics in HFmrEF

Studies on the link between HFmrEF and ncRNAs are still very limited (Savarese et al., 2022). Most reports focus on HFrEF and HFpEF, while HFmrEF is usually treated as an “intermediate phenotype” in comparative studies. In one analysis of transcriptomic data from patients with heart failure, HFmrEF was described as lying between HFpEF and HFrEF, with an ncRNA profile that may show transitional traits (Garcia-Padilla et al., 2022). So far, however, no independent and systematic research has been carried out in HFmrEF patients. Although ncRNAs are regarded as important factors in the development of heart failure, the molecular characteristics of HFmrEF remain unclear and need further confirmation through primary studies (Jalink et al., 2023).

### 4.2 ncRNA expression signatures in heart failure comorbidities and complications

**FIGURE 5:**
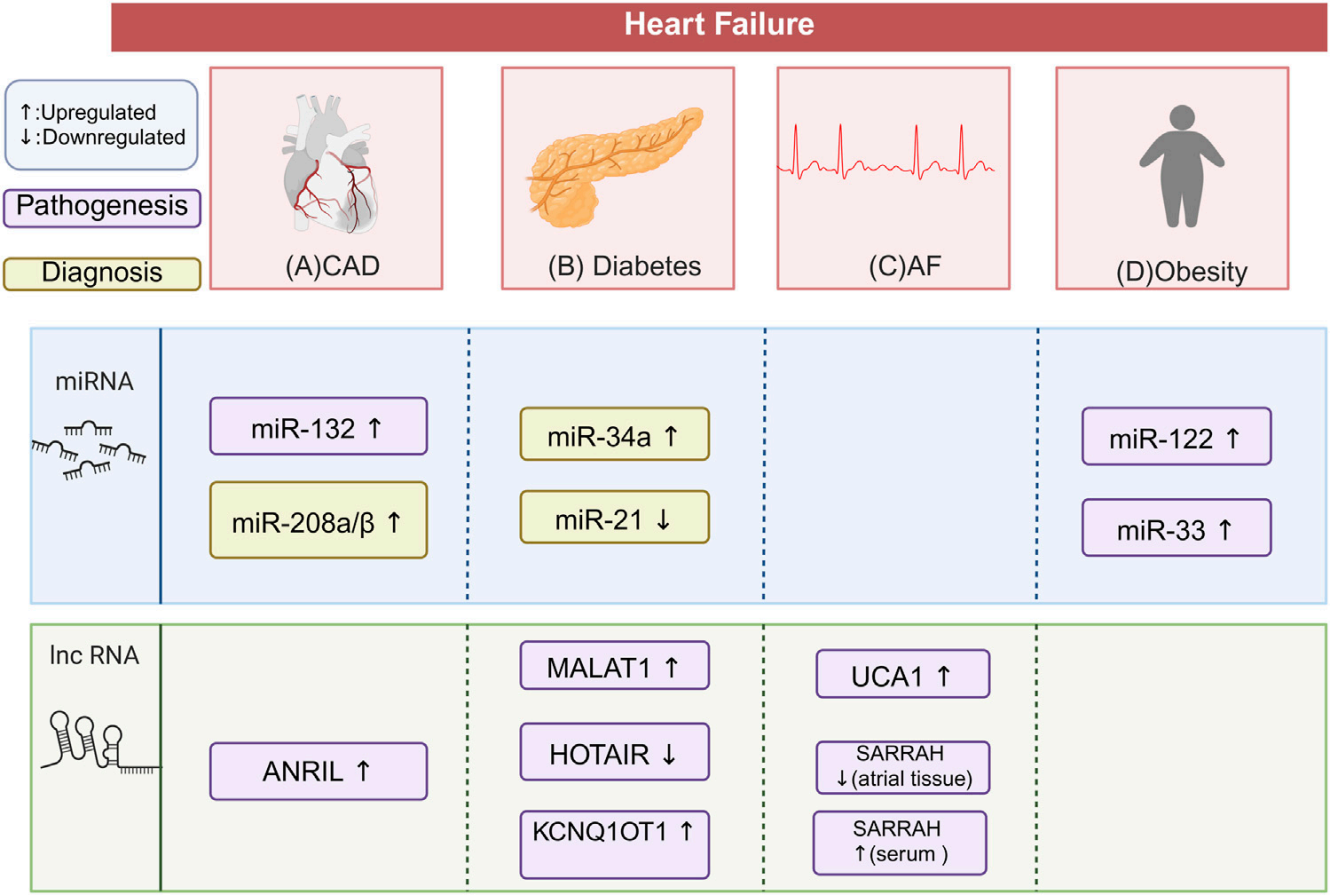
Differential ncRNA Expression in HF Comorbidities and Complications. This figure illustrates the characteristic ncRNA signatures in patients with HF and its comorbidities and complication. The potential biomarkers highlighted need to be validated through large-scale, multi-center clinical trials. This does not suggest that these biomarkers are irrelevant to the disease process. **(A)** In HF patients with CAD, miR-132 and lncRNA ANRIL are upregulated, contributing to the pathogenesis of CAD. miR-208a/β is also upregulated, helping diagnosemyocardial injury alongside troponin. **(B)** In HF patients with diabetes, miR-34a is upregulated and miR-21 is downregulated, both serving as biomarkers for HFrEF. lncRNA MALAT1 is upregulated, increasing myocardial apoptosis and fibrosis. LncRNA KCNQ1OT1 is also upregulated, promoting cardiomyocyte pyroptosis and fibrosis. lncRNA HOTAIR is downregulated, promoting cardiac oxidative stress. **(C)** In HF patients with AF, lncRNA UCA1 is upregulated, promoting myocardial hypertrophy. lncRNA SARRAH is downregulated in atrial tissue but upregulated in serum, linked to resistance to oxidative stress and ischemic injury. **(D)** In HF patients with obesity, miR-33 and miR-122 are both upregulate. miR-122 promotes cardiac hypertrophy and fibrosis. miR-33 Participates in cardiovascular remodeling. The figure is created in https://BioRender.com.

Comorbidities such as diabetes, coronary artery disease (CAD), and chronic kidney disease (CKD) are highly prevalent among patients with HF. These conditions not only significantly impact HF progression but may also accelerate its deterioration. In addition, complications such as atrial fibrillation (AF) further exacerbate disease burden and contribute to adverse outcomes. Both circulating and tissue-specific ncRNAs exhibit distinct expression profiles in these comorbid and complicated states. Within the “ncRNAs–epigenetics–cardiomyopathy” framework, these ncRNAs signatures influence disease pathways through epigenetic mechanisms (Figure 5).

#### 4.2.1 ncRNA expression signatures in heart failure comorbidities

In diabetic cardiomyopathy (DCM), lncRNA MALAT1 is significantly elevated, promoting cardiomyocyte apoptosis and fibrosis by acting as a ceRNA for miR-185-5p and activating the RhoA/Rho kinase pathway (Wang et al., 2023). Conversely, HOX Transcript Antisense Intergenic RNA (HOTAIR) is downregulated in the hearts of diabetic mice, and cardiac-specific overexpression of HOTAIR mitigates oxidative stress, thereby improving cardiac function (Qi and Zhong, 2018). In DCM models, lncRNA KCNQ1 Opposite Strand Transcript 1(KCNQ1OT1) is markedly upregulated both in high-glucose–treated cardiomyocytes and in the myocardium of diabetic mice (Yang et al., 2018). Silencing KCNQ1OT1 markedly attenuates cardiomyocyte pyroptosis and fibrosis by modulating the miR-214-3p–mediated inflammatory axis involving caspase-1 and TGF-β_1_ (Yang et al., 2018).

Additionally, in a diabetes-induced cardiomyopathy model, upregulation of miR-34a is associated with left ventricular diastolic dysfunction (Bernardo et al., 2022). However, inhibition of miR-34a did not significantly improve diastolic function (Bernardo et al., 2022). In contrast, miR-21 is downregulated (Dai et al., 2018). Reduced miR-21 expression has also been observed in HFpEF patients with type 2 diabetes, indicating diastolic dysfunction (Dai et al., 2018).

In HF patients with CKD, circulating miR-21 is significantly reduced and positively correlates with both renal dysfunction (proteinuria, eGFR decline) and the severity of heart failure (BNP levels, NYHA class) (Wang et al., 2020; Li et al., 2021).

In HF patients with CAD, miR-132 is significantly upregulated in both myocardial tissue and plasma, promoting myocardial remodeling and fibrosis (Täubel et al., 2021). Furthermore, miR-132 enhanced risk prediction for HF hospitalization beyond traditional risk factors, with a continuous net reclassification index of 0.205 (p = 0.001) (Masson et al., 2018). The elevation of miR-208a/β (myomiR) in the plasma of patients with CAD and HF reflects the degree of myocardial injury and can serve as a supplementary biomarker to troponin (Huang et al., 2021). The lncRNA ANRIL (CDKN2B-AS1) is strongly expressed in coronary atherosclerotic plaques and peripheral blood of heart failure patients. This elevated expression is significantly linked to the risk of myocardial fibrosis and unstable plaques (Fasolo et al., 2019).

Obesity, hypertension, and COPD are likewise prevalent among patients with HF and exert a substantial influence on disease progression. In obesity, metabolic dysregulation is accompanied by abnormal ncRNA expression. For instance, miR-122 is elevated in obese states and, through exosomal transfer, contributes to myocardial hypertrophy and fibrosis, while miR-33, an essential regulator of lipid metabolism, is implicated in cardiovascular remodeling (Wang et al., 2019; Ortega et al., 2023). In hypertension, alterations in the expression and function of vascular- and myocardium-enriched ncRNAs have been reported; however, their precise contribution to heart failure progression remains to be further investigated (Zhang, 2009; Dong et al., 2010). In COPD, ncRNAs associated with inflammation and oxidative stress, including miR-21 and miR-146a, have been implicated in disease mechanisms; however, direct evidence from human studies on HF–COPD comorbidity remains scarce (Sato et al., 2010; Sai et al., 2024). Overall, compared with diabetes, coronary artery disease, and chronic kidney disease, mechanistic research into these comorbidities in the context of ncRNA regulation remains underdeveloped, underscoring the need for further investigation to refine HF phenotypic stratification and mechanistic understanding (Rech et al., 2018).

#### 4.2.2 ncRNA expression signatures in high-mortality complications of heart failure

High-mortality complications, including AF, cardiogenic shock, and acute kidney injury, are common in HF patients and have a significant impact on disease progression. AF is especially prevalent, affecting more than half of those with new-onset HF, 44% of patients with acute HF, and 33% of those with chronic HF (Bidaoui et al., 2024). Research on ncRNAs expression and epigenetic mechanisms in high-mortality complications is still limited. This section will specifically examine the characteristic ncRNAs expression patterns in atrial fibrillation.

In HF patients with comorbid AF, lncRNA urothelial carcinoma-associated 1 (UCA1) is upregulated in those with atrial fibrillation compared to patients without a history of atrial fibrillation (Boichenko et al., 2025). UCA1 promotes cardiomyocyte hypertrophy and proliferation through the miR-184/miR-128 axis, thereby contributing to atrial structural remodeling (Zhou et al., 2018; Boichenko et al., 2025). The expression of lncRNA SCOT1-antisense RNA regulated during aging in the heart (SARRAH) is downregulated in the atrial tissue of patients (Ramos et al., 2023). SARRAH forms RNA–DNA–DNA triplex structures at the promoters of antioxidant and pro-survival genes (e.g., NRF2, GPC6, PDE3A), directly enhancing their transcription and conferring resistance to oxidative stress and ischemic injury in cardiomyocytes (Trembinski et al., 2020). However, compared with patients with normal sinus rhythm, the serum levels of SARRAH were increased in AF patients (Ramos et al., 2023). The clinical significance and mechanisms still need to be further investigated.

## 5 Therapeutic prospects of non-coding RNAs and epigenetic targets in heart failure

Advances in understanding the molecular basis of HF show that single-target therapies are inadequate for personalized treatment. The “ncRNAs–epigenetics–cardiomyopathy” framework guides the development of multi-target strategies, especially by modulating ncRNA–epigenetic interactions to restore cardiac structure and function (Table 4). Applying this framework creates new opportunities for HF therapy. However, a critical gap remains between preclinical evidence and its successful clinical translation, especially with respect to long-term efficacy and safety in human patient.

**TABLE 4:** Combined therapeutic strategies of ncRNAs and epigenetic factors.

ncRNA	Inhibitor target	Strategy	Function	References
miR-21	HDAC, DNMT	Inhibit myocardial fibrosis	Improvement of ventricular remodeling	Wehbe et al. (2019)
lncRNA MALAT1	EZH2	Reduce myocardial oxidative stress	Inhibition of cardiac remodeling	Levy et al. (2022)
miR-133	HDAC	Inhibit myocardial hypertrophy	Restoration of cardiac function	Wehbe et al. (2019)
lncRNA H19	EZH2	Reduce EZH2 activity	Alleviation of myocardial fibrosis	Levy et al. (2022)

### 5.1 Antisense oligonucleotides and mimic strategies targeting miRNAs

Abnormal expression of miRNAs plays a pivotal role in driving pathological changes, including cardiomyocyte hypertrophy and myocardial fibrosis. As a result, antagomirs and mimics have gained attention as promising RNA-based therapeutic options. Similarly, miR-21 antagomirs suppress fibroblast activation and reduce myocardial inflammation in preclinical models (Yang and Hu, 2019). Epigenetic modulators, such as HDAC inhibitors (e.g., Vorinostat, Trichostatin A) and DNMT inhibitors (e.g., Decitabine), are being investigated for their ability to improve myocardial fibrosis and metabolic dysfunction (Ooi et al., 2015; Han et al., 2022). However, their clinical use is limited by toxicity and off-target effects.

To enhance therapeutic specificity, combinatorial strategies integrating low-dose epigenetic drugs with targeted ncRNA interventions are gaining momentum (Song et al., 2025). For example, miR-133 mimics combined with HDAC4 inhibitors synergistically downregulate hypertrophic gene expression and reverse cardiac remodeling (Wehbe et al., 2019). Similarly, co-administration of miR-21 antagomirs with Vorinostat or Decitabine inhibits miR-21 activity, reduces fibrosis, and improves ventricular structure and function (Gorica et al., 2022).

CDR132L, an LNA-modified antisense oligonucleotide targeting miR-132, is the first RNA-based therapy for heart failure to reach clinical testing (Täubel et al., 2021). In the Phase 1b randomized, double-blind, placebo-controlled trial (NCT04045405), 28 patients with chronic ischaemic HFrEF received two intravenous doses 4 weeks apart across four escalating cohorts (0.32, 1, 3, and 10 mg/kg; 5:2 randomization per cohort) (Täubel et al., 2021). The treatment was well tolerated, induced sustained dose-dependent suppression of circulating miR-132, and in pooled patients receiving ≥1 mg/kg showed exploratory benefits, including a median 23.3% reduction in NT-proBNP, narrowing of QRS duration, and favorable trends in fibrosis biomarkers (Täubel et al., 2021).

Despite these encouraging signals, critical limitations must be acknowledged. The study was small, single-centre, and underpowered, with low placebo allocation and short follow-up; only ischaemic HFrEF patients were enrolled, limiting external validity, and no myocardial biopsies were obtained to confirm cardiac tissue engagement (Täubel et al., 2021); see also editorial perspective (Baker and Giacca, 2021). Potential selection and reporting biases further constrain interpretation, underscoring the need for larger multicentre Phase II trials with diverse HF phenotypes and clinically meaningful endpoints (Baker and Giacca, 2021; Täubel et al., 2021).

More broadly, CDR132L exemplifies the translational challenges faced by RNA therapeutics, including effective cardiac delivery, off-target interactions, long-term immunogenicity, dose optimization, and manufacturing scalability (Michel et al., 2021; Bano et al., 2022; Migliorati et al., 2022; Dzau and Hodgkinson, 2024; Obexer et al., 2024).

### 5.2 lncRNA-targeted interventions and CRISPR/dCas9-based regulation

Locked nucleic acid (LNA)-Gapped Molecule for RNA Editing (GapmeRs) have been used to degrade nuclear-localized lncRNAs, reversing their gene-silencing effects in HF models (Shah and Giacca, 2022).

The CRISPR/dCas9 system, directed by specific single-guide RNAs (sgRNAs), allows for the transcriptional repression (CRISPRi) or activation (CRISPRa) of lncRNA promoters without causing permanent DNA changes, making it a flexible and reversible tool for gene regulation (M et al., 2021).

Upregulation of lncRNA MHRT in cardiomyocytes has been shown to counter oxidative stress-induced apoptosis through Nrf2 signaling activation (Liu et al., 2023). CRISPRa represents a feasible tool to achieve such upregulation in a controllable manner. CRISPR/dCas9-based approaches have been developed to modulate EZH2/PRC2 activity at specific genomic loci (Liu et al., 2010). Separately, GapmeR-mediated knockdown of lncRNAs such as MALAT1 and H19 has been proposed as a means to mitigate cardiac injury (Levy et al., 2022). The development of CRISPR–epigenome platforms, such as dCas9–DNMT3a fusion proteins, enables targeted methylation of ncRNA loci, reconstructing “epigenetic memory” and establishing long-term therapeutic reprogramming in HF (Qian and Liu, 2024).

Despite these promising findings, several critical challenges hinder the clinical translation of CRISPR/dCas9-based epigenetic regulation. First, off-target effects remain a major safety concern, as imperfect sgRNA binding can lead to unintended gene modulation, raising the risk of unpredictable pathophysiological consequences (Kuscu et al., 2014; Wu et al., 2014). Second, efficient and cardiomyocyte-specific delivery is still problematic. Viral vectors such as adeno-associated virus (AAV) provide stable transgene expression but are limited by pre-existing immunity, packaging constraints, and potential long-term toxicity. Studies in mouse and rat models have shown that myocardial delivery of LNP–mRNA, whether by intravenous or localized administration, is feasible at the preclinical stage (Evers et al., 2022).

However, translation into standardized clinical practice remains premature. From ethical and regulatory perspectives, CRISPR/dCas9 should not be assumed a low-risk alternative to existing gene-editing tools; its safety profile depends on delivery route, timing, and indication (Huerne et al., 2022). Clinical translation further requires verification of reversibility and durability, rigorous standards for risk–benefit evaluation and informed consent, and mechanisms to prevent misuse. Stronger audit and regulatory baselines are essential across preclinical and clinical stages (Alex and Winkler, 2024).

These limitations highlight that while CRISPR/dCas9 offers unique flexibility for transcriptional and epigenetic modulation, substantial refinement in specificity, delivery, and safety frameworks will be essential before its application in human heart failure therapy.

### 5.3 Nanocarrier systems, exosomes, and clinical translation

Exosomes, with their low immunogenicity and ability to be taken up by cardiac tissue, have emerged as promising delivery vehicles. Mesenchymal stem cell-derived exosomes can deliver lncRNA MALAT1 and have been reported to exert cardioprotective effects, including anti-inflammatory and anti-apoptotic actions, in cardiac models (Zhu et al., 2023).

Nanocarrier-based delivery systems have also shown significant progress. Vehicles such as lipid nanoparticles (LNPs), polymeric microspheres (PLGA), and RNA hydrogels have been employed to deliver circRNAs with enhanced efficacy and tissue specificity in cardiac ischemia models (Yuan Z. et al., 2024). Certain circRNAs can act as miRNA sponges to regulate targets like HDAC4; similar mechanisms suggest a potential cardioprotective role for circRPAN3-like molecules in HF models (Tang et al., 2021). LNPs are well established as clinically viable RNA carriers and have been adapted for cardiovascular applications. Preclinical studies suggest that LNP-based delivery of therapeutic RNAs can modulate fibrosis and promote functional recovery, but definitive evidence for reversing myocardial remodeling in large-animal or clinical settings is still limit (Tenchov et al., 2021; Wang et al., 2022).

Several exosome-based RNA delivery platforms are currently under preclinical and early clinical development, including applications in cardiovascular regeneration. Exosome-based delivery systems are increasingly studied for their capacity to transport ncRNAs with relatively low immunogenicity and to cross critical biological barriers, including the blood–brain barrier (Alvarez-Erviti et al., 2011; Wang et al., 2018; Mentkowski and Lang, 2019; Patil et al., 2020). Engineered exosomes can even be redirected toward the heart and cardiomyocytes. Yet, translation into the clinic is still hampered by safety and manufacturing issues. A key limitation is the inconsistency of exosome production: yields and cargo loading vary considerably between batches, highlighting the urgent need for standardized, GMP-compliant protocols and well-defined critical quality attributes (Colao et al., 2018; Théry et al., 2018; Ahn et al., 2022). Furthermore, greater precision in cargo composition and targeting is essential to secure reproducible, cardiomyocyte-specific delivery (Colao et al., 2018; Wang et al., 2018; Mentkowski and Lang, 2019). Long-term safety also remains uncertain. Biodistribution data indicate predominant uptake and possible accumulation in organs such as the liver and spleen, while recent studies stress the importance of rigorous immunogenicity assessment, particularly under repeated dosing (Smyth et al., 2015; Morishita et al., 2017; Xia et al., 2024).

## 6 Conclusion

The pathogenesis of heart failure is driven by complex transcriptional and epigenetic regulation. As key regulators in HF, ncRNAs influence essential pathophysiological processes, including cardiac remodeling, inflammation, apoptosis, and metabolic reprogramming, by orchestrating epigenetic mechanisms such as DNA methylation, histone modifications, and chromatin remodeling (Greco and Condorelli, 2015). In this review, we present an integrative working model that explicitly links ncRNAs, epigenetic mechanisms, and cardiomyopathy along a unified axis. Unlike prior models that considered these regulators in isolation, our framework integrates multilayer interactions across HF subtypes, comorbidities, and complications, thereby offering a dynamic and testable roadmap for translational research.

Although advances have been made, significant gaps remain in our understanding of the interplay between ncRNAs, epigenetics, and cardiomyopathy. The available profiling of ncRNAs is still fragmentary, particularly for patients with HFmrEF, those with multiple comorbidities, and subgroups stratified by sex or age. Biomarker candidates identified to date often fail to reproduce consistently across different biospecimen types, show insufficient disease specificity, and are frequently reported from small, single-center studies. Addressing these limitations will require harmonized detection standards, validation across large multicenter cohorts, and systematic assessment in diverse human populations to narrow the translational divide.

In terms of therapeutics, initial proof-of-concept efforts such as the CDR132L clinical trial and experimental exosome-based delivery platforms illustrate the potential of ncRNA-directed strategies. Nevertheless, translation into practice remains hampered by modest patient numbers, incomplete validation across relevant tissues, and broader challenges that include ensuring cardiac-specific uptake, minimizing off-target effects, addressing long-term immune responses, achieving scalable production, and navigating regulatory frameworks. Likewise, CRISPR/dCas9-based approaches, while conceptually promising, are still constrained by underdeveloped delivery vehicles and unresolved safety and ethical concerns. Exosome-based systems may provide a more physiologically compatible delivery route, but critical barriers remain in terms of GMP manufacturing standards, lot-to-lot reproducibility, biodistribution mapping, and demonstration of durable safety.

To address these limitations, future research should focus on key areas. Multicenter validation studies are needed to assess the efficacy and safety of ncRNA-based therapies across diverse heart failure populations, enabling broader use of these treatments (Fumarulo et al., 2025). Additionally, machine learning models that integrate multi-omics data could improve AI-based biomarker prediction. This would help identify ncRNA regulatory roles in heart failure progression and treatment response, leading to more personalized therapies (DeGroat et al., 2024; Asta et al., 2025). Furthermore, optimizing delivery systems, such as lipid nanoparticles and exosomes, will improve the specificity, stability, and targeting of RNA-based therapies, ensuring safe and effective delivery to cardiac tissue (Hou et al., 2021). Finally, comprehensive ncRNA profiling, particularly in underexplored groups such as patients with mid-range ejection fraction (HFmrEF) and those with common comorbidities, will be critical for refining molecular stratification and advancing individualized treatment strategies (Spinetti et al., 2023; Paterek et al., 2024; Parvan et al., 2025).

By integrating mechanistic insights with translational barriers and research priorities, our framework moves beyond existing models. It provides not only a coherent conceptual axis but also a realistic and expandable platform to guide multi-targeted, individualized therapies in heart failure.

## Glossary

AAV: adeno-associated virus
AF: atrial fibrillation
AUC: Area Under the Curve
BNP: B-type natriuretic peptide
CAD: coronary artery disease
CaSR: calcium-sensing receptor
CeRNAs: competing endogenous RNAs
Chaer: Cardiac Hypertrophy Associated Epigenetic Regulator
CKD: chronic kidney disease
CircRNAs: circular RNAs
DCM: diabetic cardiomyopathy
DNMTs: DNA methyltransferases
DOX: Doxorubicin
EF: ejection fraction
FOXO3: Forkhead box O3
GAS5: Growth Arrest Specific 5
GapmeRs: antisense oligonucleotides
HATs: histone acetyltransferases
HDACs: Histone deacetylases
HF: Heart failure
HFrEF: heart failure with reduced ejection fraction
HFmrEF: heart failure with mid-range ejection fraction
HFpEF: heart failure with preserved ejection fraction
HIPK3: Homeodomain-Interacting Protein Kinase 3
HOTAIR: HOX Transcript Antisense Intergenic RNA
H3K27me3: histone H3 at lysine 27
H3K4me3: histone H3 at lysine 4
KCNQ1OT1: KCNQ1 Opposite Strand Transcript 1
KLF3-AS1: Kruppel-like factor 3 antisense RNA 1
LNA: locked nucleic acid
LncRNAs: long non-coding RNAs
LNPs: lipid nanoparticles
LVEF: left ventricular ejection fraction
MALAT1: Metastasis-Associated Lung Adenocarcinoma Transcript 1
MHRTs: RNA transcripts
MiRNAs: microRNAs
MiRs: microRNAs
m6A: N6-methyladenosine
MIRI: myocardial ischemia-reperfusion injury
NcRNAs: non-coding RNAs
NT-proBNP: N-terminal pro-B-type natriuretic peptide
Nt: nucleotidy
P: P-value
PLGA: polymeric microspheres
ROS: Reactive Oxygen Species
SARRAH: SCOT1-antisense RNA regulated during aging in the heart
SgRNAs: single-guide RNAs
SNHG8: Small Nucleolar RNA Host Gene 8
SRA1: steroid receptor RNA activator 1
TGF-β1: transforming growth factor-β1
TUG1: taurine upregulation 1
UCA1: Urothelial Carcinoma Associated 1
XIST: X-inactive specific transcript
ZNF609: Zinc Finger Protein 609
