# Correction: Non-coding RNAs in heart failure: epigenetic regulatory mechanisms and therapeutic potential

**DOI:** 10.3389/fgene.2025.1746339

**Published:** 2025-11-24

**Authors:** Yubo Ren, Bomeng Zhao, Luo Lv, Jingyuan Yang, Xiangting Nan, Bao Li, Bin Yang

**Affiliations:** 1 Second College of Clinical Medicine, Shanxi Medical University, Taiyuan, Shanxi, China; 2 The First College of Clinical Medicine, Shanxi Medical University, Taiyuan, Shanxi, China; 3 Shanxi Medical University, Taiyuan, Shanxi, China; 4 Department of Cardiology, The Second Hospital of Shanxi Medical University, Taiyuan, China; 5 School of Medicine, Shanxi Medical University, Taiyuan, China

**Keywords:** heart failure, non-coding RNA, epigenomics, microRNAs, long non-coding RNA, circular RNA, therapeutics

There was a mistake in Figure 4 as published. In Figure 4, the word “faiure” was incorrectly spelled. The correct spelling is “failure.” The corrected Figure 4 appears below.

**FIGURE 4:**
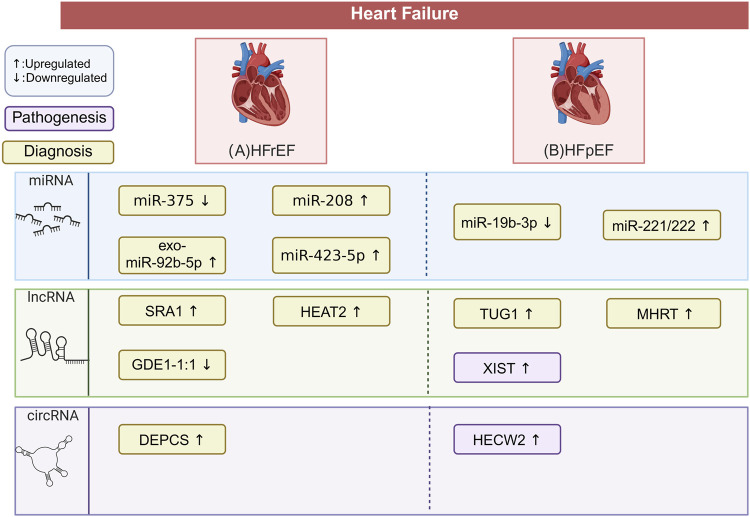
Differential ncRNA Expression in HF Types. This figure outlines the characteristic ncRNAs signatures across in patients with different types of HF. The pathogenesis and diagnoses depicted are all related to heart failure. The potential biomarkers mentioned require validation through large-scale, multi-center clinical trials. This does not imply that these biomarkers are without impact on the disease pathogenesis. **(A)** In HFrEF, miR-375 and lncRNA GDE1-1:1 are downregulated, while miR-208, miR-423-5p, exo-miR-92b-5p, lncRNA SRA1, lncRNA HEAT2, and circRNA DEPCS are upregulated. These molecules are potential biomarkers for HFrEF diagnosis. **(B)** In HFpEF, miR-19b-3p is downregulated, while miR-222/221, lncRNA TUG1, lncRNA MHRT, and circRNA HECW2 are upregulated. These molecules serve as potential biomarkers for HFpEF, with lncRNA XIST and circRNA HECW2 linked to profibrotic and inflammatory pathways. The figure is created in https://BioRender.com.

There was a mistake in Figure 5 as published. In Figure 5, the words “faiure” and “diabete” were incorrectly spelled. The correct spellings are “failure” and “diabetes,” respectively. The corrected Figure 5 appears below.

**FIGURE 5:**
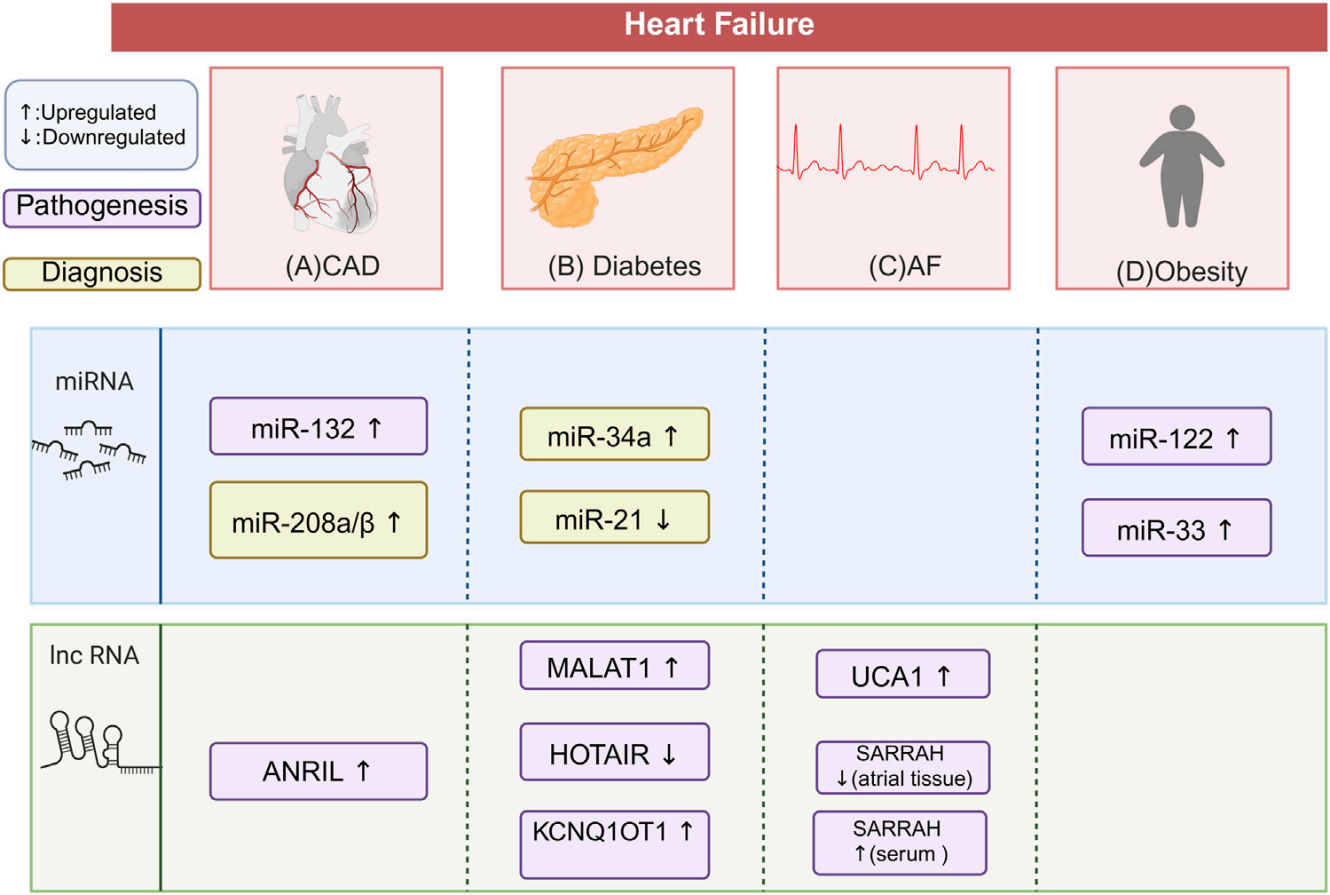
Differential ncRNA Expression in HF Comorbidities and Complications. This figure illustrates the characteristic ncRNA signatures in patients with HF and its comorbidities and complication. The potential biomarkers highlighted need to be validated through large-scale, multi-center clinical trials. This does not suggest that these biomarkers are irrelevant to the disease process. **(A)** In HF patients with CAD, miR-132 and lncRNA ANRIL are upregulated, contributing to the pathogenesis of CAD. miR-208a/β is also upregulated, helping diagnose myocardial injury alongside troponin. **(B)** In HF patients with diabetes, miR-34a is upregulated and miR-21 is downregulated, both serving as biomarkers for HFrEF. lncRNA MALAT1 is upregulated, increasing myocardial apoptosis and fibrosis. LncRNA KCNQ1OT1 is also upregulated, promoting cardiomyocyte pyroptosis and fibrosis. lncRNA HOTAIR is downregulated, promoting cardiac oxidative stress. **(C)** In HF patients with AF, lncRNA UCA1 is upregulated, promoting myocardial hypertrophy. lncRNA SARRAH is downregulated in atrial tissue but upregulated in serum, linked to resistance to oxidative stress and ischemic injury. **(D)** In HF patients with obesity, miR-33 and miR-122 are both upregulate. miR-122 promotes cardiac hypertrophy and fibrosis. miR-33 Participates in cardiovascular remodeling. The figure is created in https://BioRender.com.

The original article has been updated.

